# Incidence of BRAF V600E Gene Mutation Among Lebanese Population in Melanoma and Colorectal Cancer: A Retrospective Study Between 2010 and 2019

**DOI:** 10.7759/cureus.29315

**Published:** 2022-09-19

**Authors:** Rim Masri, Amani Al Housseiny, George Aftimos, Nizar Bitar

**Affiliations:** 1 Internal Medicine, Lebanese University Faculty of Medicine, Beirut, LBN; 2 General Medicine, Lebanese University Faculty of Medicine, Beirut, LBN; 3 Anatomical Pathology, Institut National de Pathologie, Beirut, LBN; 4 Hematology and Medical Oncology, Sahel General Hospital, Beirut, LBN

**Keywords:** colorectal cancer, melanoma, oncogene, braf v600e mutation, incidence

## Abstract

Introduction: Cancers arise owing to the accumulation of mutations in critical genes that leads to uncontrolled cell division and the avoidance of apoptosis. Among these oncogenes, BRAF is a potent mitogen-activated protein kinase (MAPK) pathway activator known to be somatically mutated by a glutamic acid to valine substitution at codon 600 (V600E). It is a common finding in various types of human cancers, including malignant melanoma and colorectal cancer (CRC), and is considered a poor prognostic factor and a predictive biomarker. The study aims to determine the incidence of BRAF V600E gene mutation in Lebanese patients with melanoma and CRC and its correlation with gender and age.

Methods: We conducted a retrospective cohort design study in which 210 and 132 patients diagnosed to have melanoma and CRC, respectively, were recruited from 2010 to 2019 from "L’Institut National de Pathologie," where a specific polymerase chain reaction is used to detect BRAF mutations. Data from digitized records were collected, including demographic characteristics (age and gender), cancer type, and BRAF mutation. The collected data were analyzed using SPSS Statistics version 20.0 (IBM Corp., Armonk, NY). A p-value < 0.05 was considered significant.

Results: The incidence of BRAF mutation in melanoma is 88.10%. There is female predominance with a ratio of 2.6:1 (p = 0.240) and the majority of patients aged between 40 and 60 years (51.2%) with a mean age of 53.74 years. While in CRC, BRAF is mutated in 7.5% with a ratio of 1.2:1 of male predominance (p = 0.999). The majority of patients (54.8%) were between the ages of 60 and 80 years, with a mean age of 65.5 years.

Conclusion: BRAF is a frequent oncogenic mutation that is found in lethal tumors. Targeted therapies for these cancers interfere with developing more effective therapeutic strategies, which affect the treatment response in BRAF mutants and improve the prognosis of the patients.

## Introduction

B-type rapidly accelerated fibrosarcoma (BRAF) kinase gene mutation is a B-viral oncogene that was first discovered to be somatically mutated in 2002 and seen in up to 15% of many human malignancies [1], including 40% to 80% of all types of malignant melanoma [2,3] and 9% to 15% of colorectal cancers (CRCs) [4], as well as many other tumors and some inflammatory diseases. It is an independent negative prognostic factor that demonstrates a growing incidence and mortality rate. BRAF is the most potent protein kinase that activates the intracellular signaling cascade of the mitogen-activated protein kinase (MAPK) pathway, involved in the control of cell growth, proliferation, migration, and the avoidance of programmed cell death [2]. The transversion of thymine to adenine at nucleotide position 1799 (T1799A), which results in the substitution of valine (GTG) by glutamic acid (GAG) at codon 600 of exon 15, in the activation loop of the kinase domain, designated the BRAF V600E mutation [1]. This mutation occurs in most events and accounts for more than 90% of all BRAF mutations [5]. These changes increase the activation of catalytic activity of BRAF kinase up to 700-fold [6], and its ability to stimulate constitutively the entire MAPK cascade [7].

Malignant melanoma

Melanoma is the most aggressive skin cancer that arises in the melanocytes of the lower part of the epidermis, with great mortality rates and a high potential for dissemination. To treat the patient effectively, its accurate identification and subsequent diagnosis are essential. Normally, BRAF is preferred to be used to activate the MAPK cascade by cAMP-dependent kinase signaling (RKIP) [8]. Otherwise, in BRAF-mutated melanocytes, the expression of RKIP is reduced [9], the cell invasion is enhanced, in addition to a higher ulceration rate, shortened survival rate, and a worse prognosis is caused compared to patients whose disease expressed wild-type BRAF [10]. The BRAF V600 mutation is the only validated predictive biomarker for patients with melanoma, which is highly predictive for response to BRAF inhibitors with a low rate of primary resistance [11], resulting in longer median survival than those patients with wild-type BRAF melanoma. BRAF-mutated melanoma has been found to be positively linked to the melanocortin-1 receptor genetic variants [12], and it is frequently associated with intermittent sun-exposed skin, but rarely in uveal melanomas [13,14].

Colorectal cancer

CRC is one of the most prevalent types of cancer, and the presence of BRAF mutation is strongly associated with the sporadic high microsatellite instability (MSI-H) in about 29% to 33% [15], resulting in a very poor prognosis. The median overall survival achieved by BRAF V600E-mutated patients is about 11.4 months, compared to 43 months in BRAF wild-type tumors [16]. The BRAF V600E-mutated CRC may frequently demonstrate the hyper-methylation of the DNA CpG island methylator phenotype (CIMP), inducing the inactivation of a tumor suppressor gene known as the human mutL homolog 1 (MLH1) that is involved in DNA mismatch repair (MMR). This results in the deficiency of the MMR system and consequently the development of the MSI-H phenotype [17,18].

BRAF-mutated tumors, which are a well-known target of genetic mutations in the tumorigenesis of human cancer, are most commonly treated by the BRAF-targeted small molecule inhibitors. Several generations of RAF inhibitors were developed to be used as single agents or in combination. Such inhibitors, vemurafenib and encorafenib, are used in the treatment of advanced stages of BRAF V600E-mutated malignant melanoma and CRCs, respectively [19,20].

Overall, the incidence and the pathological features of this mutation in clinical practice, including the relationship between the mutation status and the efficacy of the available BRAF inhibitors, especially among Lebanese populations, still have limited data. In view of the paucity of our knowledge and with the aim to partially cover this gap, the present study is the first report of a retrospective cohort study that represents the incidence of BRAF V600E gene mutation in malignant melanoma and CRC development and its correlation with sex and age, within the Lebanese population by using real-time polymerase chain reaction (PCR) technique at the pathologic department with a view to modifying the therapeutic approach and follow-up.

## Materials and methods

Study design

This was a retrospective study about the incidence of BRAF V600E gene mutation conducted among Lebanese patients having melanoma and CRC between 2010 and 2019, recruited from “L’Institut National de Pathologie” (INP) in Beirut, a medical center grouping specialists in anatomical and cytological pathology in Lebanon.

Study population

The sample size was 210 for melanoma and 132 for CRC, including all eligible subjects. Regarding the selection of the study subjects, all Lebanese patients with one of these cancers (melanoma or CRC), with wild-type or mutated BRAF gene, and with completed clinicopathologic data during the study period were included in the study. Non-Lebanese citizens were excluded. The diagnosis of the cases was confirmed by a specific PCR used to detect BRAF gene mutations, especially the V600E mutation.

Data collection

Medical charts were reviewed by employing a consistent dataset to collect data related to demographic characteristics (age and gender), the type of cancer, and the type of mutation. Basic data are collected from the patient files available in INP. The study received ethical approval from the Institutional Review Board (IRB) at INP. All the information collected has been fully anonymized to ensure patient confidentiality.

Statistical analysis

The data were obtained from the database and classified based on the RAF mutational status (mutated or wild-type), the type of mutation, the gender, and the age of the patients. Statistical analysis was performed using SPSS Statistics version 20.0 (IBM Corp., Armonk, NY) and results were described using frequencies, percentages, means, and median age. Absolute numbers and percentages were used to report categorical variables. Chi-square distribution was employed to determine the association between categorical variables. A p-value < 0.05 was considered significant.

## Results

Melanoma

Incidence of BRAF

A total of 210 Lebanese melanoma cancer patients were registered between 2010 and 2019 from the INP center in Beirut. Using the real-time amplification refractory mutation system (ARMS)-PCR and sequencing PCR products by the technicians of the pathology center, mutations were found in 185/210 (88.10%) melanoma cases. All mutations were in BRAF V600E form with the absence of any other types of RAF mutations. Other samples showed a normal wild-type genotype that accounted for 25 out of 210 (11.90%) cases (Table 1).

**Table 1:** The frequency of RAF mutational status (mutated vs. wild-type) in melanoma and CRC between 2010 and 2019. CRC: colorectal cancer.

Results	Melanoma (2010-2019)			CRC (2017-2019)		
	Mutated	Wild-type	Total	Mutated	Wild-type	Total
Frequency	185	25	210	10	122	132
Percentage	88.10	11.9	100	7.5	92.5	100

BRAF and Gender

Among patients known to have BRAF-mutated melanoma cancer, 64% were females and 24.1% were males, with a female predominance of 2.6-fold. At the 95% significance level, the relationship between BRAF mutation and gender was statistically insignificant since the p-value was 0.240 (>0.05). However, BRAF mutations were not detected in about 7.1% of female melanoma patients compared to 4.8% of males (Table 2).

**Table 2:** The distribution of BRAF mutation (mutation vs. wild-type) according to the gender of the patients having melanoma and CRC. CRC: colorectal cancer.

		Melanoma			CRC		
		Wild-type	Mutated	Total	Wild-type	Mutated	Total
Gender	Male	10 (4.80%)	51 (24.10%)	61 (28.90%)	71 (54.00%)	6 (4.20%)	77 (58.20%)
	Female	15 (7.10%)	134 (64.00%)	149 (71.10%)	51 (38.50%)	4 (3.30%)	55 (41.80%)
	Total	25 (11.90%)	185 (88.10%)	210 (100.00%)	122 (92.50%)	10 (7.50%)	132 (100.00%)

BRAF and Age

**Figure 1:**
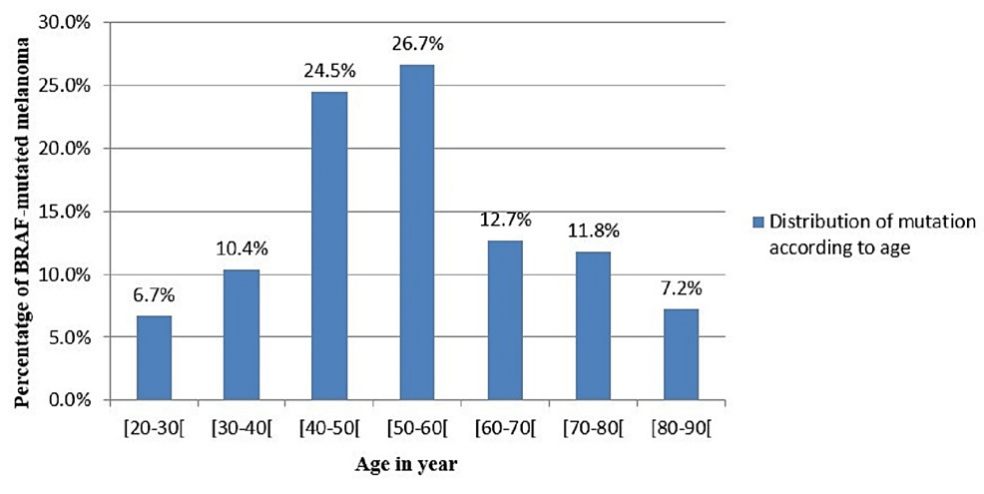
Distribution of BRAF mutation according to age in melanoma.

In melanoma cancer, the incidence of BRAF mutation increases with age. The majority of patients found were between the ages of 40 and 60 years (51.2%), with a minority of younger patients aged 20 to 30 years (6.7%) and those older, aged 80 to 90 years (7.2%). The mean age was 53.74 years and the median age was 55 years (range: 20-90 years) (Figure 1). Thus, the highest incidence of BRAF-mutated melanoma was found in patients of middle age, particularly those whose range was between 50 and 60 years old.

Colorectal cancer

Incidence of BRAF

Over the time from 2017 to 2019, 132 Lebanese CRC patients were recruited from the INP center during this period and included in the study. There were 7.5% (10 out of 132) of patients with mutated-BRAF oncogene and 92.5% (122 out of 132) with wild-type genes (Table 1).

BRAF and Gender

Among the patients diagnosed to have CRC, there were 58.2% males and 41.8% females with a male predominance ratio of 1.2:1. The proportion of female patients with mutated CRC was 3.3%. While BRAF mutation was detected in about 4.2% of male cases. At a 95% significance level, the relationship between BRAF mutation and gender was statistically extremely insignificant since the p-value was 0.999 (>0.05). Nevertheless, a total of 92.5% of patients were found to have BRAF-negative forms consisting of 38.5% of women and 54% of men (Table 2).

BRAF and Age

**Figure 2:**
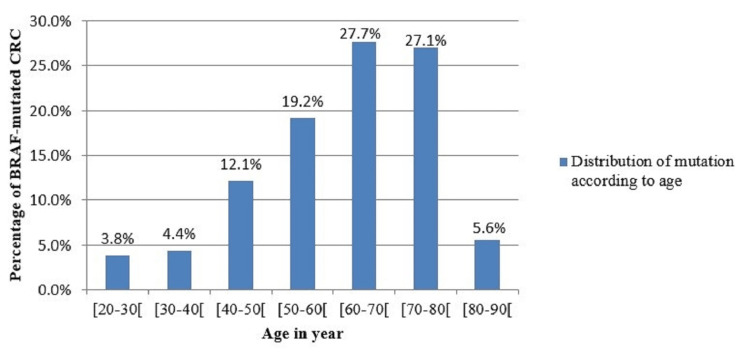
Distribution of BRAF mutation according to age in CRC. CRC: colorectal cancer.

Overall, the incidence rates of BRAF-mutated CRC also increased with age. Where 54.8% of BRAF-mutated patients were 60 to 80 years old with the age of the majority being between 60 and 70 years old (27.7%), compared to those younger than 60 years old (39.5%) and a minority aged between 20 and 30 years (3.8%). The mean age was 65.5 years and the median age was 55 years (range: 20-90 years) (Figure 2).

## Discussion

The incidence of BRAF gene mutation is specific for each tumor and varies by geographic location [3]. In our study, BRAF gene mutation was detected in 88.10% of patients having melanoma; this is the highest incidence compared to the global incidence found in the literature (40-80%) of melanoma harboring BRAF gene mutation [2,3].

This 88.10% is contradictory to a previously published study in Lebanon in 2013, which showed an incidence of 49% of BRAF mutation in patients having melanoma [21]. The differences between the two studies could be attributed to the sample size. In our study, 210 patients diagnosed to have melanoma were recruited, whereas the sample size is reduced approximately to half in the study done in 2013, where only 95 patients were included. In addition, concerning the method used for the detection of BRAF mutation, its sensitivity may differ between the pathologic center of each study and that may explain the difference; both studies used PCR. In our study, 185 of 210 patients were detected to have BRAF gene mutation and 25 patients have wild-type BRAF gene, but in another study, 45 of 95 patients were found to have BRAF mutation, whereas 42 of 95 patients have wild-type BRAF gene while eight of 95 patients have failed PCR [21].

Melanoma genesis is a complicated process. The difference among the incidences is affected by several factors. First, chronic sun and ultraviolet (UV) exposures [22] cause damage to the DNA in skin cells, especially intermittent to intense exposure to the skin. Second, people with fair skin and less pigment (melanin), blond or red hair, and light-colored eyes have less protection from damaging UV radiation [23]. A previous study showed different incidences of BRAF mutation in melanoma around the world [24].

In this study, the incidence of BRAF mutation in melanoma reaches its maximum in patients aged between 40 and 60 years (24.5% between 40 and 50 years and 26.7% between 50 and 60 years). However, the incidence was lower in patients younger than 40 or older than 60 years. And this result is explained by the accumulated sun exposure with age. While some studies reported no correlation between the BRAF mutation and the age or the gender of the patient [25], several studies around the world have reported higher mutation rates in younger patients and explained that the cumulative minor UV exposure during childhood causes the appearance of melanoma in young patients [22].

Concerning CRC, the incidence of BRAF oncogene mutation in CRC varies worldwide. The incidence in our study was 7.5%, which is close to the incidence observed across the globe (9%) [4], but higher than the incidence found in a study in the Indian population, which showed a much lower incidence of 4.7% [26]. Literature noted that the highest rates of CRC are present in developed countries, such as Australia, New Zealand, Europe, and North America. On the other hand, the lowest rate of CRC is found in Western Africa and Asia [27].

The variation in the incidence of BRAF mutation in patients diagnosed to have CRC is due to the different methods of analysis of BRAF mutation (for example, the use of fresh, frozen, formalin-fixed, paraffin-embedded (FFPE), biopsies, and PCR), and demographic variation in the population studied. Lifestyle and dietary habits play important role in the causation of CRC. Obesity, a sedentary lifestyle, alcohol, processed meats, a red-meat-rich diet, and smoking [28] are all factors found in developed countries that increase the risk of developing BRAF gene mutation and having CRC. On the other hand, the incidence may descend in some countries due to the early detection and removal of precancerous polyps as a result of CRC screening and the impact of the reduction of its risk factors [29].

However, older patients are at increased risk of developing CRC: the incidence of the mutation in our study was higher in patients aged between 60 and 80 years (27.7% between 60 and 70 years and 27.1% between 70 and 80 years) and lower in patients younger than 60 or older than 80 years. Moreover, in our study, the incidence of male patients with mutated BRAF gene was higher than female patients with a ratio of 1.2 approximately (4.2% versus 3.3%, respectively). CRC develops in older people because of their prolonged exposure to carcinogens and substances in the food we eat that may cause mutations as a result of random errors when a cell’s DNA is copied before it divides. As a result, the cells accumulate more mutations the longer we live [30].

Study strengths

The data are collected from INP, which receives samples from a wide range of hospitals all over Lebanon. This can make our population diverse during a wide time range between 2010 and 2019.

Study limitations

This study faced some limitations. First, the sample size in our study is very small: 210 patients for melanoma and 132 for CRC. Second, the coronavirus disease 2019 pandemic was an obstacle that stopped us from collecting the data, particularly concerning the age of the patients from INP, and then analyzing it statistically. And finally, our study was retrospective, which limits the analysis of variables that could better discriminate between the prognosis, such as the lack of information about treatment, outcomes, and their clinicopathological correlation.

Study perspectives

Our study aims to find the incidence of BRAF gene mutation in patients having melanoma and CRC among the Lebanese population. It is important to find this incidence of BRAF mutation in other types of cancer. The detection of BRAF oncogenic mutation was the first step to determine the proportion of patients that may benefit from BRAF inhibitors targeted therapy to start the correct treatment, to shed light on the most effective regimens, to decrease the costs and the side effects caused by maltreating wild-type melanoma and CRC by BRAF inhibitors, and to improve the quality of life, decrease mortality, and increase the overall survival of patients. It is important to follow up with the patients concerning the therapy, outcomes, and its clinicopathological measures. Thus, a longer follow-up period in a large cohort study is required to identify a possible correlation of BRAF mutation with clinical outcomes of disease recurrence, prognosis, and survival. In addition, in detecting the BRAF mutation, it is important to specify which type of mutation among the BRAF gene is detected to improve the efficacy of therapy by BRAF inhibitors in targeting the pathway that is malfunctioning. This is important to ensure the correct initiation of BRAF inhibitors treatment.

## Conclusions

In conclusion, the BRAF V600E mutation is frequent among patients diagnosed to have melanoma, and it has a very low incidence of CRC in the Lebanese population. The study of the mutation is a process that takes several years and needs multiple study groups to understand its impact on tumorigenesis and its response to targeted therapy or the pathway related to the resistance mechanism. Due to BRAF inhibitors resistance, further studies should be done to understand the molecular mechanism of resistance and to find mutations other than BRAF that are implicated in these cancers to facilitate the development of upcoming-generation inhibitors and improve the patient’s prognosis, survival, and clinical outcomes. This should be an interesting topic of research in the future.
